# Combined Albenazole-Praziquantel Treatment in Recurrent Brain Echinococcosis: Case Report

**Published:** 2019

**Authors:** Tomislava SKUHALA, Vladimir TRKULJA, Mislav RUNJE, Mirjana BALEN-TOPIĆ, Dalibor VUKELIĆ, Boško DESNICA

**Affiliations:** 1. University Hospital for Infectious Diseases “Fran Mihaljeviæ”, Zagreb, Croatia; 2. Department of Pharmacology, School of Medicine, Zagreb University, Zagreb, Croatia; 3. TAPI Research and Development Analytics, Pliva Croatia, Zagreb, Croatia

**Keywords:** Albendazole, Praziquantel, Brain echinococcosis, Albendazole-sulphoxide concentration

## Abstract

We present a 40-year-old woman with a history of relapsing echinococcosis who had undergone a number of surgical procedures for cyst removal (right pulmectomy, cardiac surgery and 6 subsequent brain surgeries and two gamma knife procedures) and was admitted to University Hospital for Infectious Diseases “Fran Mihaljeviæ”, Zagreb, Croatia in 2014 for pre-operative medical treatment of brain hydatidosis in the right parietal region. We aimed to attain a high cyst albendazole sulphoxide (ASO) concentration in order to achieve a more pronounced protoscolex inactivation and a high serum ASO concentration (reflecting the tissue concentrations) to reduce the risk of disease recurrence. The patient was treated with a higher dose of albendazole (15 mg/kg/day for 4 wk) that we had found effective in patients with liver hydatidosis, and combined with praziquantel over the last 14 d at a dose that is typically used to treat neurocysticercosis with an intention to improve ASO bioavailability. Neither serum nor cerebrospinal fluid concentrations on day 10 apparently differed from those on day 24 indicating a lack of an effect of praziquantel on ASO bioavailability. Intra-cystic ASO concentration was below the lower limit of quantification, but above the limit of detection. After the 7^th^ episode of the disease and combined albendazole-praziquantel and surgery treatment, the patient achieved a 3-year remission. With the apparent lack of a meaningful pharmacokinetic praziquantel-albendazole interaction, this is most likely ascribable to the use of a higher albendazole dose than previously.

## Introduction

Echinococcosis is an anthropozoonosis caused by the larvae of *Echinococcus granulosus* (1). In humans, the parasite develops in the form of hydatid cyst(s) mostly affecting the liver or the lungs (1–3). Cerebral cysts are present in 2%–3% of neurologically symptomatic patients typically invading the middle cerebral artery territory, but any location is possible and prevalence of asymptomatic brain infection is unknown. Symptoms depend on the location and may vary from simple headache to uncal herniation (4). Treatment of the brain hydatid cysts includes surgical removal and treatment with benzimidazole anthelmintic drugs. The pharmacological mono treatment is indicated in cases of surgically unreachable cysts, but it is typically adjunctive to surgery (4).

Praziquantel, a broad spectrum anthelmintic ineffective against *E. granulosus* (5), by an unknown mechanism increases bioavailability of albendazole-sulfoxide (ASO), the active metabolite of albendazole and combination of the two is superior to albendazole alone in patients with intra-abdominal hydatid cysts (6–9).

Here we present a patient with brain hydatidosis who experienced a number of recurrent episodes despite the peri-operative treatment with albendazole, and who eventually underwent a combined albendazole-praziquantel peri-operative treatment to achieve a prolonged remission but without apparent effect of praziquantel on bioavailability of ASO.

## Case report

Written informed consent was obtained from the patient for publication of this case report and any accompanying images.

**Fig. 1:**
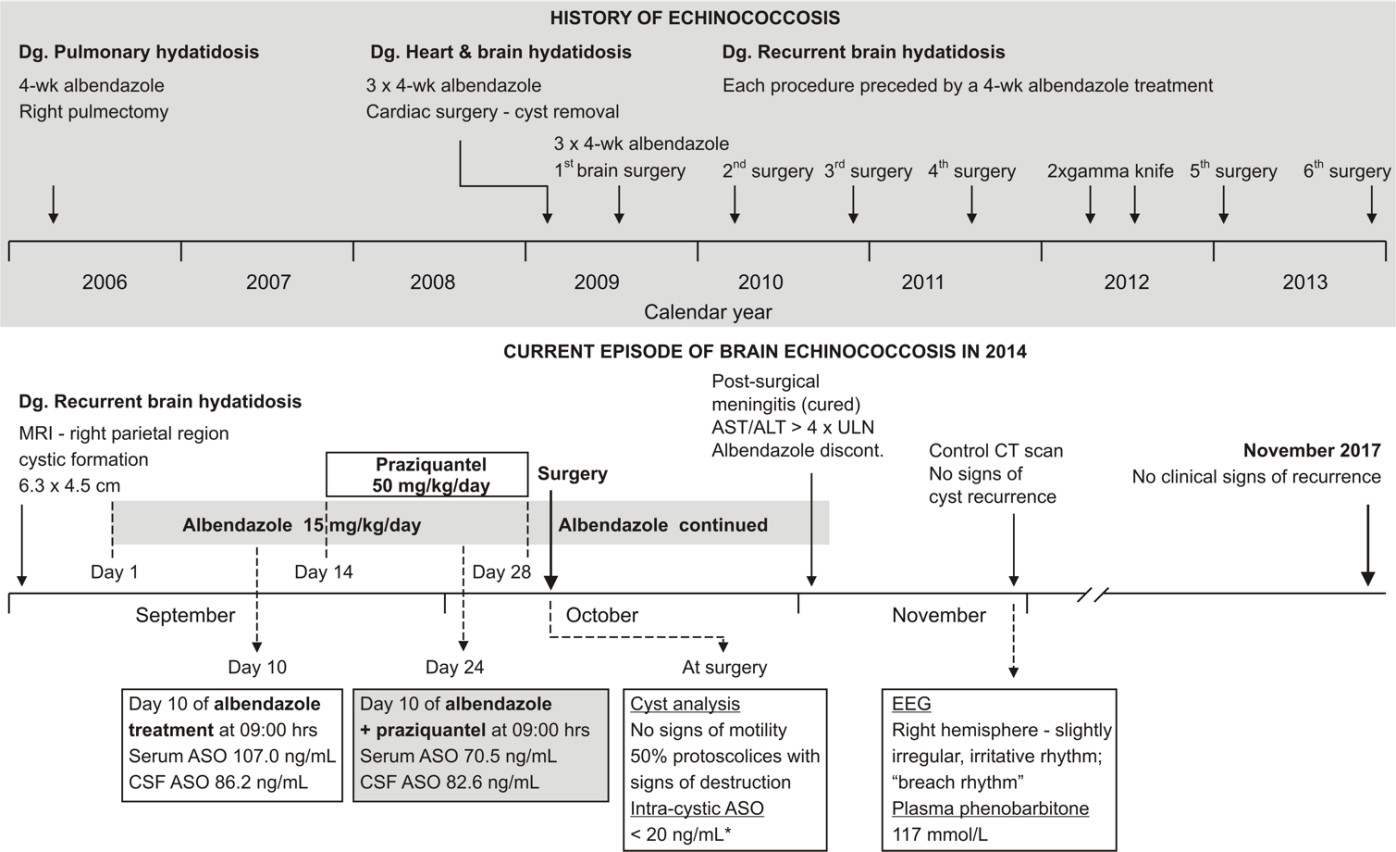
Schematic outline of the patient's echinococcosis history (upper panel) and sequence of treatments, evaluations and outcomes of the current episode (lower panel). ALT – alanine aminotransferase; ASO – albendazole-sulphoxide; AST – aspartat aminotransferase; CSF – cerebrospinal fluid; CT – computed tomography; EEG – electroencephalography; MRI – magnetic resonance imaging; ULN – upper limit of normal *The lower limit of quantification of the method was 20 ng/mL and the lower limit of detection was 2.0 ng/mL. The measured value could not be precisely quantified since it was between the two values

In Sep 2014, a 40-year-old woman with a history of relapsing echinococcosis was referred to the University Hospital for Infectious Diseases, Zagreb, Croatia for pre-operative medical treatment of brain hydatidosis since magnetic resonance imaging revealed a conglomerate of cystic formation (6.3 × 4.5 cm) in the right parietal region. Since 2006, when she was first diagnosed with pulmonary hydatidosis, she had undergone a number of surgical procedures for cyst removal–right pulmectomy in 2006, cardiac surgery and first brain surgery in 2009, and then 5 subsequent surgeries and two gamma knife procedures for brain hydatidosis by the end of 2013 (Fig. 1). Each procedure was preceded by albendazole treatment.

On admission, she complained of headaches, forehead paresthesias, dysgraphia, dyslexia, anxiety, instability and loss of peripheral vision. Apart from a decreased nasolabial fold prominence on the left side and increased gamma glutamiltranspherase (GGT) (104 U/L), the results of physical examination and of laboratory tests were unremarkable. The only chronic treatment was methylphenobarbitone 200 mg/day started in 2009, after the first brain surgery.

A 28-day albendazole treatment was commenced: 15 mg/kg/d split into 3 daily administrations with a fatty meal to increase bioavailability. At day 14, praziquantel 50 mg/kg/day (three times daily, together with albendazole) was added for the subsequent 14 d. Within 20 h after the last dose, she underwent craniotomy and cyst removal (Fig. 1). Albendazole was continued post-operatively, as well. Peripheral venous blood samples and cerebrospinal fluid (CSF) samples were taken on days 10 and 24 (day 10 of combined albendazole + praziquantel) of pharmacological pre-treatment at the time of the expected peak (3 h after the morning dose) (6,7) for determination of ASO concentrations. Intrasurgically, a sample of the cyst content was taken for the same purpose, and another sample was taken for microbiological evaluation. ASO concentrations were measured by high-pressure liquid chromatography method with UV detection (10). Neither serum nor CSF concentrations on day 10 (albendazole-only treatment) apparently differed from those on day 24 (day 10 of combined treatment) indicating a lack of an effect of praziquantel on ASO bioavailability (Fig. 1).

**Fig. 2:**
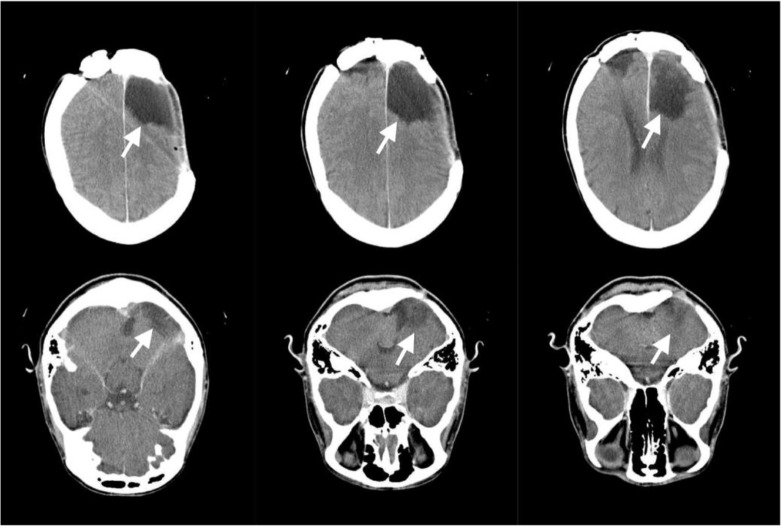
Axial native and postcontrast computer tomography (CT) scan at the parietal lobe level. An 5.6x3.7 cm encephalomalacic area is seen in the right parietal region, as a consequemce of previous cystic lesion conglomerate extirpation. Encephalomalacic lesions in the right cerebellar hemisphere and the left parietal lobe did not change in comparison with previous images performed. There are no neuroradiologic signs of echinococcosis recurrence

Intra-cystic concentration was below the lower limit of quantification (20 ng/mL), but above the limit of detection (2.0 ng/mL). Un-stained wet mount confirmed echinococcosis, whereas further analysis (samples incubated with sodium taurocholate in Hank's buffer for 48 h at 37 °C to re-create conditions of the canine gastrointestinal system “stimulation” (10)) revealed lack of viable protoscolices, whereas 50% of protoscolices showed signs of destruction. Subsequently (Fig. 1), she developed post-surgical bacterial meningitis which resolved upon a 14-day meropenem and vancomycin treatment. At that time rise of aspartate aminotransferase (89 U/L), alanine aminotransferase (143 U/L), GGT (464 U/L) and alkaline phosphatase (136 U/L) was observed and albendazole was discontinued (Fig. 1). One week after resolution of meningitis, a computed tomography brain scan revealed no signs of echinococcosis recurrence with a lesion at the sight of the previous cyst location (Fig. 2). Neurological evaluation was unremarkable and plasma phenobarbitone concentration (117 mmol/L) was within the therapeutic range (60–170 mmol/L). In Nov 2017, 3 years after the index craniotomy, the patient is without signs of disease recurrence.

## Discussion

All fourteen 4-week treatments implemented prior to surgical procedures before the index surgery used the most common albendazole dosing schedules (10 mg/kg/day twice daily) yet the disease relapsed after each procedure. Therefore, the present episode was treated with a higher dose of albendazole (15 mg/kg/day in 3 administrations) that we found effective in patients with liver hydatidosis (10), and was combined with praziquantel over the last 14 d at a dose typically used to treat neurocysticercosis (5) with an intention to improve ASO bioavailability (11,12).

Upon ingestion, albendazole is presystemically rapidly oxidized to ASO] mainly via cytochrome P450 (CYP) 3A4], which is further oxidized to inactive sulphone (by CYPs) (13). Patients with brain hydatidosis are maintained on antiepileptic drugs, typically carbamazepine, phenytoine or (methyl) phenobarbitone, all of which induce CYP3A4 and reduce systemic ASO concentrations (by around 65% in the case of phenobarbitone) (13), but increased ASO bioavailability by praziquantel is seen in patients with CYP induction, as well (11). In this patient, praziquantel did not increase systemic or CSF availability of ASO, which is in line with the reported high inter-individual variability of the praziquantel-albendazole interaction (13). The intra-cystic ASO concentration appeared low, much lower than reported for non-brain cyst locations (10, 14), but according to the microbiological analysis, it was sufficient to inhibit viability and induce a considerable destruction of protoscolices. Although the observed 3-year remission does not exclude a possibility of a further disease relapse, it is the longest disease-free period in this patient since the initial occurrence of brain hydatidosis in 2009. With apparent lack of a meaningful pharmacokinetic praziquantel-albendazole interaction, it is most likely ascribable to the use of a higher (15 mg/kg/day) albendazole dose than in the previous cycles.

## Conclusion

Brain echinococcosis is a rare localization of echinococcosis, there are no available data of ASO concentration in brain cysts, no potential positive drug interactions for treatment of this location of cysts and optimal treatment for long remission, and therefore further studies are needed.
